# Intermittent Fasting in Youth: A Scoping Review

**DOI:** 10.21203/rs.3.rs-4524102/v1

**Published:** 2024-07-01

**Authors:** Jomanah A. Bakhsh, Alaina P. Vidmar, Sarah-Jeanne Salvy

**Affiliations:** Department of Population and Public Health Sciences, University of Southern California; Children's Hospital Los Angeles and Keck School of Medicine of USC, Department of Pediatrics, Center for Endocrinology, Diabetes and Metabolism; Department of Biomedical Sciences, Cedars-Sinai Medical Center

**Keywords:** Intermittent fasting, pediatrics, pediatric obesity, time restricted eating, alternate day eating

## Abstract

Intermittent fasting (IF) focuses on the timing of eating rather than diet quality or energy intake, with evidence supporting its effects on weight loss and cardiometabolic outcomes in adults. However, there is limited evidence for its efficacy in adolescents and emerging adults. To address this, a scoping review examined IF regimens in individuals aged 10 to 25, focusing on methodology, intervention parameters, outcomes, adherence, feasibility, and efficacy. The review included 39 studies with 731 participants aged 15 to 25. Methodologies varied, with 18 studies on time-restricted eating and others requiring caloric restriction. Primary outcomes included cardiometabolic risk factors (11/29), body composition (9/29), anthropometric measurements (8/29), and feasibility (2/29). Most studies reported significant weight loss. This review underscores IF's potential in treating obesity in this age group but highlights the need for rigorous studies with standardized frameworks for feasibility to ensure comparability and determine IF’s practicality in this age group.

## 1.0 Introduction

From 1999 to 2018, class I obesity increased from 16–21% in youth ages 12 to 19 years of age, while severe obesity (classes II and III) rose from 5–8% [1]. This is concerning as obesity has been linked to comorbidities including type 2 diabetes, sleep apnea, and fatty liver disease [2–4]. In January 2023, the American Academy of Pediatrics (AAP) issued new clinical practice guidelines for managing obesity in young people. The AAP's guidelines recommend prompt, multifaceted intervention strategies, including intensive lifestyle and behavioral interventions, obesity pharmacotherapy, and bariatric surgery [4].

Adherence to treatment recommendations is possibly the strongest predictor of weight loss [5–8], and the best strategy for a given individual is the one they are willing and able to practice and sustain. Therefore, there is a critical need to diversify interventions to ensure young people can identify the strategies that work for them based on personal preference, developmental and social stage, and obesity phenotype. Intensive family-based health and behavioral interventions are the cornerstone of pediatric obesity treatment. However, these interventions can only be effective in improving long term health outcomes if they are delivered and received as intended. While family-based interventions work for some youth and their families who are able and willing to engage and adhere to all the required treatment recommendations, a meaningful proportion disengage prematurely. For many adolescent and emerging adults, a family-based approach that relies on involvement of caregivers may not best fit the needs and new autonomous roles of adolescents and emerging adults. Furthermore, modifications in the home food environment and complex behavior changes may not be practical or sustainable during this developmental period [4]. Developing and testing novel intervention approaches can diversify our treatment toolkit to help a greater segment of young people to achieve their health goals [9–12].

Dietary regimens focusing on *s* food is consumed, rather than on the quality or quantity of food consumed [13, 14] have gained popularity in the last decade. Intermittent fasting (IF) involves altering the timing and duration of eating and fasting periods [15]. Common IF regimens include the 5:2 diet (eating ad libitum 5 days per week and fasting for two non-consecutive 24-hour periods), alternate-day fasting (typically involves alternating a day of eating ad libitum with a fast day), time-restricted eating (TRE, required fasting for a specified period of time each day), and religious fasting [16, 17]. IF interventions emerged from findings indicating that, while diet quality and exercise are beneficial for health, the timing of eating may independently predict health outcomes and disease risks [18]. Spreading eating events across the day (i.e., > 14 h eating window) and late-night eating have been linked to poor cardiometabolic health in adults.^87–89^ By contrast, well-timed eating and fasting, such as eating earlier in the day and fasting in the evening and night, has been shown to induce weight loss, enhance insulin sensitivity, improved sleep quality, and decrease inflammation [17, 19–27]. In adults, TRE studies indicate that shortening the eating period can lead to a 10–25% reduction in energy consumption, even without intentional caloric restriction [19–22]. In addition, cycling between periods of fasting and eating has also been linked to reduced markers related to aging, diabetes, autoimmune diseases, cardiovascular health, neurodegenerative conditions, and cancer [28–30]. IF has also been shown to modulate inflammatory responses and reduce oxidative stress [22, 24–27]. Given the role of inflammation and oxidative stress in accelerating aging, exploring this effect from an early stage in life is compelling.

IF might be appealing to adolescents and emerging adults in allowing more freedom around food choices and because of its simplicity [14, 26]. Yet, studies of IF have mainly focused on adults over the age of 30, and research in youth is still in its early stages [31, 32]. Since 2019, over 50 reviews have summarized the effectiveness of IF in adults [33–86], with no comparable published summary among adolescents and emerging adults [31, 32]. This scoping review seeks to address this gap in examining published IF studies in children, adolescents, and emerging adults. With the goal of highlighting gaps in knowledge and informing future trials, specific considerations were given to: (1) methodology; (2) intervention parameters including eating timing, duration, and additional components; (3) adherence monitoring; (4) feasibility assessment; (5) primary and secondary outcomes captured; (6) potential iatrogenic effects; and (7) effects on anthropometric, metabolic outcomes, and markers of biological aging.

## 2.0 Methods

### 2.1 PRISMA-ScR Framework.

A PRISMA-ScR scoping review was conducted. Consistent with the PRISMA framework [87, 88] the following steps were executed: (1) identify the research question by clarifying and linking the purpose and research question; (2) identify relevant studies by balancing feasibility with breadth and comprehensiveness; (3) select studies using an iterative team approach to study selection and data extraction; (4) chart the data incorporating numerical summary and qualitative thematic analysis; and (5) collate, summarize and report the results, including the implications for practice and research.

### 2.2 Identifying the Initial Research Questions [89].

The focus of the review was to investigate IF interventions that included adolescent and emerging adults. To ensure that a wide range of relevant studies was captured, the following two-part research question was crafted to guide the search: In IF interventions that include adolescents and emerging adults: 1) What were the intervention parameters, adherence monitoring approaches and fidelity measures employed and primary and secondary outcomes captured, and 2) What is the reported feasibility and efficacy?

A structured search was applied utilizing the PubMed bibliographic database. The first author (JAB) created the initial PubMed search strategy using a combination of Medical Subject Headings (MeSH) and keywords for intermittent fasting, health effects, and youth. The search was restricted to studies published since 2000, on humans, who are adolescents or young adults, and written in English. Intermittent fasting included time-restricted eating, intermittent energy restriction, and alternate day fasting interventions. Team members (JAB, SJS, and APV) reviewed the strategy and preliminary results to modify and improve the search strategy. With the team's approval, JAB customized the search using controlled vocabulary and keywords in the database listed above. The search strategy included the following terms: intermittent fasting OR time restricted eating OR time restricted feeding OR alternate day fasting OR intermittent energy restriction OR meal timing OR eating window AND weight OR metabolism OR metabolic OR body composition OR aging OR inflammation OR inflammatory OR oxidative stress AND youth OR young adult OR adolescent. All resulting citations were exported into a Mendeley library, and duplicates were removed. No additional efforts were conducted to seek out grey literature, including other study registries, websites, or conference proceedings. On March 31, 2024, the search was repeated in the bibliographic database to identify any more recent studies.

### 2.3 Study Selection.

Titles and abstracts were first screened, and then eligible full-text articles were screened by one author (JAB) (title abstracts: n = 240, full-text n = 91). For the initial screening of abstracts, the inclusion criteria were as follows: (1) articles are in English; (2) included participants with age equal to or less than 25 years old; (3) report of a primary or second outcome that relates to a change in weight (body mass index (BMI), BMI z-score, weight in excess of the 95th percentile[%BMIp95], percent weight change), a change in metabolic markers (body composition, glycemic biomarkers, or lipid profiles), or a change in biological aging markers (inflammatory markers and oxidative stress). There was only one exclusion criterion. Studies on Ramadan IF were excluded due to its unique cultural and ceremonial context, which entails significant alterations in eating habits, sleep cycles, and often leads to increased consumption of high-sugar and high-fat foods, rendering its effects incomparable to other forms of health-promoting fasting [15]. No exclusion criteria were applied to sample size or location. During subsequent full-text screening, the independent reviewers ensured the following criteria were met for all retrieved studies: (1) publication included full text and (2) publications were peer-reviewed. Ineligible reports included dissertations, conference abstracts and proceedings, unpublished protocols, and commentaries or opinion pieces.

### 2.4 Data Charting.

Before data charting, the authors discussed the various variables and reached agreement in terms of which variables were extracted based on the scope of the review. One reviewer (JAB) developed a data-charting form to determine which variables to extract and charted the data. Then, two reviewers discussed the results, and continuously updated the data-charting form. The data extracted included sample descriptions, methodology, outcome measures, assessments, and results of interventional studies. Next, one extractor reviewed all the articles and formed the table (JAB). An additional team member double-checked the extracted data and helped revise the table (APV). Data tables facilitated analysis. Participant characteristics across trial studies, interventions, measures, and results are summarized in Tables 1 and 2.

### 2.5 Ethical Considerations.

#### Ethical approval

was not sought for this review as it relies on already published work. Additionally, this review was not registered in PROSPERO, the international database for systematic reviews in health and social care, due to the fact that scoping reviews do not fulfill the registration requirements (https://www.crd.york.ac.uk/prospero/#aboutpage).

## 3.0 Analytic Analysis

For each interventional study on the health effects of IF, the following data were abstracted: number of participants, ages of participants, study design, intermittent fasting regimen, additional intervention components, adherence monitoring method, feasibility assessment, primary and secondary outcome measures. The other included studies were analyzed conceptually, without charting of specific data.

## 4.0 Results

### 4.1 Study Selection.

**Figure 1:**
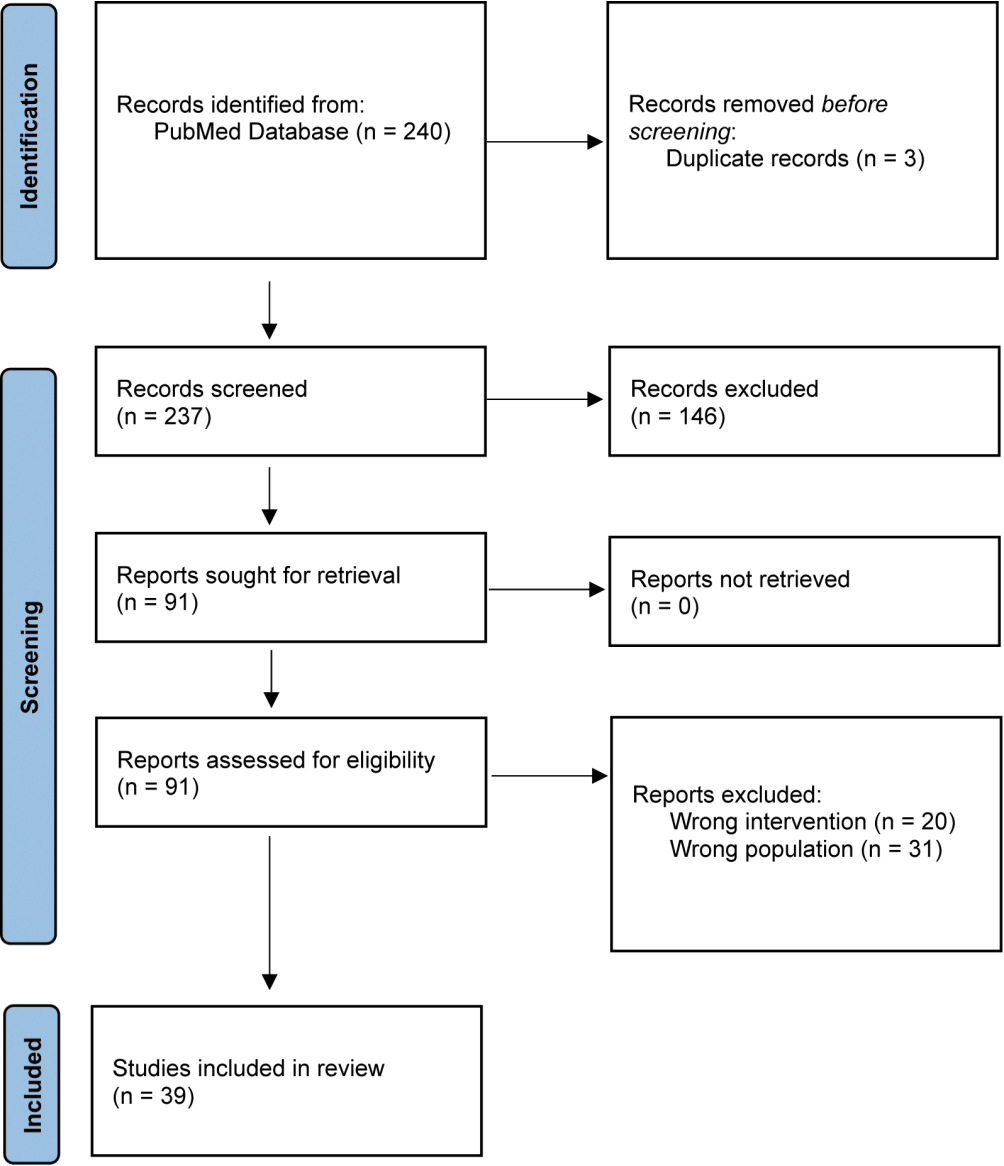
Scoping review flow diagram. Caption: the PRISMA flow diagram for new systematic reviews detailing the database searches, the number of abstracts screened, and the full texts retrieved.

**Figure 2:**
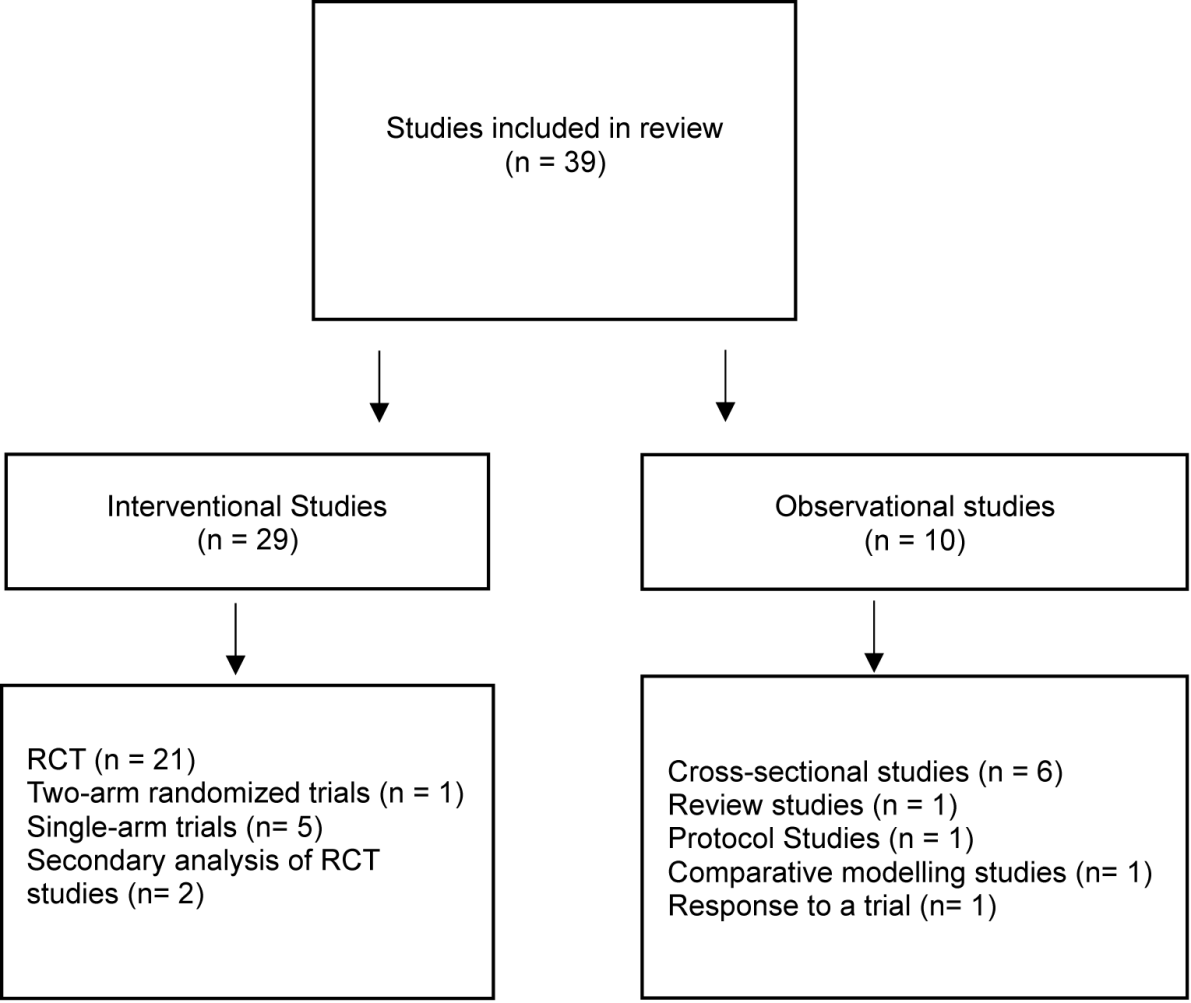
Methodologies of the Included Studies in the Scoping Review.

Upon removal of duplicates, 237 titles and abstracts were screened for eligibility. A total of 91 underwent full-text review. Of those, 39 studies met the pre-specified inclusion criteria. A PRISMA flow diagram detailing the database searches, the number of abstracts screened, and the full texts retrieved is illustrated in Figure 1. The study designs and methodologies of the included studies are cataloged in Figure 2. Of these studies, twenty-nine were interventional studies that focused on the efficacy of IF on weight, metabolism, and/or biological aging markers. Table 1 summarizes the key characteristics of the 29 interventional studies that included efficacy data, emphasizing intervention components, execution, feasibility measures, adherence monitoring, and primary and secondary outcomes. The remaining ten studies were observational studies focusing on evaluating the effects, acceptability, and implications of timing of eating within the context of weight management interventions, cardiometabolic risk factors, and eating behaviors in adolescents and young adults, without involving the experimental manipulation of timing of eating.

### 4.2 Participants.

Studies predominantly focused on adolescents and young adults, with mean ages between 15 to 25 years old [31,32,90–132]. Some included a majority of female participants (e.g., 64% in Vidmar et al. 2021) [31], while others only included male participants (e.g., 100% males in McAllister et al. 2020 and Harder-Lauridsen et al. 2017) [114,124]. A few studies reported an even sex distribution [116]. Participants' baseline weight status also varied widely, from those with a median or mean weight indicating overweight or obesity (e.g., median weight=101.4 kg in Vidmar et al. 2021) [31] to those with participants in the normal weight range (e.g., mean BMI = 22.7 kg/m^2^ in Park et al. 2021) [112].

### 4.3 Study Design Characteristics.

Thirty-nine studies were included in this review. Ten studies did not involve interventional design, but rather examined the relationships between meal timing and weight management outcomes, cardiometabolic risk factors, and eating behaviors in adolescent and emerging adults [90–98,102,105]. Twenty-nine were interventional studies that focused on efficacy or effectiveness of IF interventions on weight, metabolism, and biological aging markers, as summarized in Table 1 [31,32,107–131]. The primary outcomes varied, but primarily involved assessing the efficacy of various intermittent fasting interventions on body weight, body composition, cardiometabolic health markers, energy balance, and specific physiological responses such as glycemic control and muscle damage indicators. Secondary outcomes were also diverse and included assessments of dietary intake quality, physical activity, sleep patterns, eating behaviors, quality of life, glycemic control, blood biomarkers, microbial diversity, muscular performance, hunger, craving, mood, cognitive function, appetite, and energy intake responses. Most interventional studies involved interventions with short duration, spanning 4 to 12 weeks, which limits conclusions about long-term efficacy and safety. Among these studies, twenty-one were RCTs [31,107,109–111,113–116,120,121,123,124,126], five were single-arm trials [32,108,112,122,125], and one was two-arm randomized trial [114]. Eighteen studies utilized an 8-hour time-restricted eating window [31,95,107,108,111,112,114,115,119,121–123,126], and two tested other forms of IF, including Alternate-Day Calorie Restriction [124] and Protein-Sparing Modified Fast [125]. Other studies investigated different TRE windows or other IF protocols. For instance, Zhang et al. 2022 compared early (7:00 a.m. - 1:00 p.m.) and late (12:00 p.m. - 6:00 p.m.) 6-hour TRE windows [113], and Bao et al. 2022 tested the efficacy of a 5.5-hour TRE window compared to an 11-hour eating control group [118].

In total, eight studies involved multicomponent interventions combining TRE, continuous glucose monitoring (CGM), resistance training (RT), energy restriction, low carbohydrate and added sugar diets, brisk walking, high-intensity exercise, antioxidant supplementation, and protein-sparing modified fasts (PSMF). Keenan et al. 2022 compared continuous energy restriction with 5:2 intermittent fasting, where the IF group consumed normal calories for 5 days and significantly reduced calories on 2 days of the week [116]. Only one study conducted a comparative analysis between early and late TRE, providing unique insights into how the timing of eating windows within IF regimens can affect metabolic health, weight loss, and potentially other well-being markers [113]. The caloric requirements varied across studies, with some implementing isocaloric conditions (maintaining the same caloric intake) [107,114], energy restriction (e.g., 25% calorie deficit, very low-calorie diets) [120], and intermittent fasting days such as alternate-day fasting (ADF) [32] and intermittent energy restriction (IER) [120]. Specific interventions like the protein-sparing modified fast (PSMF) had defined caloric intake ranges (1200–1800 calories with low carbohydrate and high protein) [125].

### 4.4 Catalog of Feasibility Measures and Adherence Monitoring

To evaluate the acceptability and feasibility of healthcare interventions, a generic, theoretically grounded questionnaire was previously developed around the constructs of the Theoretical Framework of Acceptability (TFA) [133]. This tool was designed to measure seven specific elements related to feasibility: affective attitude, burden, ethicality, intervention coherence, opportunity costs, perceived effectiveness, and self-efficacy. This versatile questionnaire can be customized to analyze the acceptability of various healthcare interventions across diverse settings. Studies were evaluated on whether they measure acceptability and feasibility consistent with TFA. Table 2 presents an overview of the interventional studies, cataloged by the results reported. There was great heterogeneity in how feasibility was defined across various study designs. None of the studies utilized the seven TFA components. A few of the individual components of the framework were captured: 7/29 affective attitude, 4/29 burden, 0/29 ethicality, 0/29 intervention coherence, 0/29 opportunity costs, 1/29 perceived effectiveness, and 0/29 self-efficacy.

### 4.5 Summary of Clinical Trials

#### Body Weight

There was a variety of weight status and body composition measures utilized across reports making comparison of the effect of IF on body weight challenging. Most studies highlighted significant weight loss among participants adhering to IF protocols, though with varying degrees of weight reduction across studies and samples [31,107,108,112–115]. Vidmar et al. (2021) examined the efficacy of late TRE in adolescents with obesity. All groups experienced weight loss, with 31% of the participants of the TRE plus continuous glucose monitoring (CGM) group, 26% of the TRE with blinded CGM, and 13% of the control group [31]. Hegedus et al. (2023) reported a significant decrease BMI at the 95^th^ percentile (%BMIp95) at week 12, with a 46% reduction observed in the late TRE (lTRE) group compared to 21% in the control group with an extended eating window [107]. Zhang et al. (2022) observed decreases in weight and BMI in both early and late TRE groups compared to controls [113].

In Moro et al. (2020) study, the TRE group experienced a 2% weight change from baseline, while this was not the case for participants assigned to the control group [115].

Park et al. (2021) documented significant weight loss among female participants, while no significant weight loss was observed among male participants [112]. In contrast, research examining the combination of TRE with resistance training (RT) offers a different perspective [109,110]. Tinsley et al. (2019) investigated the effects of an 8-hour TRE combined with β-hydroxy β-methylbutyrate (HMB) supplementation and RT in active females, only to find an increase in body weight across all groups [110]. Similarly, a study by Tinsley et al. (2017) on a 4-hour TRE regimen coupled with RT in men reported no significant change in body weight [109], indicating that the efficacy of TRE on weight loss might be influenced by factors such as biological sex, baseline weight, and exercise regimens.

#### Cardiometabolic Risk Factors

Several studies reported improvements in markers of glucose metabolism [107,112,113]. For instance, Hegedus et al. (2023) found reductions in hemoglobin A1c (HbA1c) and alterations in C-peptide levels in late TRE groups [107]. Kim & Song (2023) observed reductions in fasting blood glucose and improvements in HOMA-IR, indicating better glucose regulation and insulin sensitivity [122]. Zhang et al. (2022) highlighted a decrease in insulin resistance [113]. One study also reported reductions in systolic and diastolic blood pressure [114]. Conversely, two studies [32,109] observed no metabolic changes compared to baseline. Another study reported significant reductions in fasting insulin, acyl ghrelin, and leptin concentrations during energy deprivation compared to energy balance. Postprandial hormone responses, including insulin, GLP-1, and PP, were elevated after energy deprivation, while acyl ghrelin was suppressed, indicating that altered sensitivity to appetite-mediating hormones may contribute to the adaptive response to negative energy balance [105].

McAllister et al. (2020) and Zhang et al. (2022) noted decreases in body mass and fat mass (FM) in participants adhering to TRE, while preserving lean mass [113,114]. Additionally, IF was associated with decreased liver enzymes aspartate aminotransferase (AST) and alanine transaminase (ALT) in two studies [107,111]. McAllister et al. (2020) reported increases in high-density lipoprotein (HDL) and variations in low-density lipoprotein (LDL) and total cholesterol depending on the type of TRE (ad libitum vs. isocaloric) [114]. Zeb et al. (2020) found decreased total cholesterol (TC) and triglycerides (TAG), and an increase in HDL post-TRE [111]. However, divergent effects on lipid profiles were observed as well, with increases in HDL [111,114] as well as in LDL [112,113].

#### Biological Aging Markers

Only a few studies measured markers associated with biological aging [111,113–115]. McAllister et al. (2020) and Moro et al. (2020) both reported an increase in adiponectin levels in participants following an 8-hour TRE regimen, whether combined with an ad libitum diet or an isocaloric diet. Elevated adiponectin levels are inversely associated with obesity and oxidative stress and correspond to improved metabolism and resting energy expenditure [114,115]. Additionally, Moro et al. (2020) observed a significant decrease in the neutrophil-to-lymphocyte ratio, an inflammatory marker, within the TRE groups compared to controls, indicating reduced inflammation [115]. Zeb et al. (2020) observed reductions in serum IL-1B and TNF-a levels post-TRE, though these changes were not statistically significant, suggesting a potential trend towards reduced inflammation that warrants further investigation [111]. Zhang et al. (2022) reported that superoxide dismutase (SOD), a crucial antioxidant defense in nearly all living cells exposed to oxygen, significantly increased in participants who engaged in early TRE compared to those in late TRE and control groups [113].

### 4.6 Summary of Observational Studies

Observational studies varied in their focus. Some addressed parental interest in time-restricted eating (TRE), while others looked into nutritional adequacy, concerns, and the efficacy of TRE [90–94,96]. Tucker et al. (2022) found that two-thirds of parents with children in pediatric weight management programs showed interest in time-limited eating (TLE) for ≤12 hours per day, with interest waning for stricter limits of ≤10 or ≤8 hours [90]. Lister et al. (2020) challenged the notion that continuous energy restriction (CER) is the sole method for weight management in metabolically unhealthy adolescents, proposing intermittent energy restriction (IER) as a viable alternative in tertiary settings [91]. Similarly, Lister (2017) emphasized the need for careful consideration of nutritional adequacy in energy-restricted diets, highlighting that various eating patterns can achieve both nutritional adequacy and energy restriction, which is crucial when prescribing diet interventions for adolescent weight loss [92]. Nevertheless, skipping breakfast was associated with increased cardiometabolic risk factors in adolescence, as observed is a cross-sectional survey study by de Souza et al. (2021) [93]. One study reported that diets low in carbohydrates and those involving intermittent fasting were linked to increased disordered eating behaviors, including binge eating and food cravings. These findings suggest that such restrictive diets may heighten cognitive restraint, leading to an upsurge in food cravings. However, this study's reliance on a cross-sectional design and a web-recruited university sample, predominantly female, introduces potential biases [102].

One review study evaluated the impact of the timing and composition of food intake, physical activity, sedentary time, and sleep on health outcomes, suggesting that these factors independently predict health trajectories and disease risks. This underscores the need for a unifying framework that integrates time-based recommendations into current health guidelines for children and adolescents [94]. However, the practical implications of IER, such as the risk of fostering restricted eating patterns and inhibiting growth in adolescent girls on a 600–700 kcal diet, raise concerns. Vanderwall et al. (2020) pointed out that physical activity, an essential strategy for preventing obesity and metabolic syndrome, was not adequately measured in some studies, despite its likely contributory impact. These findings collectively highlight the potential benefits and challenges of dietary interventions like TLE, CER, and IER, emphasizing the importance of ensuring nutritional adequacy and integrating physical activity for effective adolescent weight management [96].

Observational research examining the relationships between the timing of eating, weight management outcomes, and cardiometabolic risk factors suggests there is no meaningful impact on body composition. However, there may be benefits to cardiometabolic health from adopting earlier and shorter eating windows [97,98,132]. These findings are consistent with studies in adults indicating that aligning meal consumption with circadian rhythms can enhance metabolic outcomes [134,135]. One possible explanation for the disparate findings across clinical trials and observational studies is that existing observational studies have failed to consider how eating timing interacts with eating window duration to influence health. Studies in adults have reported that eating late in the day, even with shorter eating window, can worsen postprandial glucose levels and b-cell responsiveness or confers no health benefit [136]. More studies are needed to better characterize the joint influence of eating timing, eating frequency, and daily eating duration on health outcomes.

## Discussion

This scoping review catalogs published studies of intermittent fasting interventions in young people up to age 25. The review included 39 studies and revealed that there is a great heterogeneity in study design, methodology, feasibility measures, adherence monitoring, and intervention components across studies of IF in adolescents and young adults. The diversity of methodologies and outcomes makes it challenging to summarize overall efficacy of IF.

While IF interventions have the potential to be a feasible and acceptable treatment approach for adolescents and young adults, the current results highlight the need for rigorous studies to investigate feasibility of novel interventions, such as IF, utilizing standardized theoretical frameworks for acceptability to allow for comparability across studies and cohorts. As highlighted in the results; the majority of the studies included captured one to three of the seven recommended components associated with the acceptability framework however none utilized all seven components in their entirety. In addition, the majority captured this data via self-report and open-ended questionnaire with very little qualitative data to drive conclusions regarding feasibility and acceptability of IF interventions in this age group.

Furthermore, the intervention components investigated varied significantly. This was not only found among what form of IF intervention was studied but what additional components of the intervention were included. It very well may be that IF based interventions can act as a synergistic intervention to other multicomponent health and behavior approaches but the studies cannot truly be compared for efficacy when the interventions are not similar in their components. Each IF approach may be uniquely suited to a specific individual’s preferences, life stage, and resources. Thus, large, well-designed feasibility and efficacy trial should be performed for each IF approach compared to a control arm that is standardized across study designs to allow for comparability. In pediatric practice, investigators may consider utilizing a multidisciplinary, family-based intervention model given that is the most utilized health and behavioral lifestyle intervention implemented in this age group.

Time restricted approaches were the most commonly studied form of IF included in this review. Even among TRE interventions there remains much opportunity for diversity in the approach which effects outcomes. As show in the results, the timing of the eating window varied by study design with the majority allow for a participant identified eating window followed by an afternoon/evening window. This variation in study design emphasizes an important mechanistic component of IF research across all age groups regarding the underlying mechanism that results in improvement in weight and cardiometabolic risk. There remains debate as to which eating window is most preferred by participant as well as which eating window results in the greatest improvement in weight and cardiometabolic outcomes when adhered to well. Further research is needed to understand both of these questions and allow for the mechanistic discover underling IF interventions as well as the pragmatic approach to how to actually disseminate this type intervention in a real-life setting to optimize engagement and thus sustained efficacy[97].

Given that adherence to treatment recommendations is the strongest predictor of weight loss; rigorous adherence monitoring is needed in the assessment of novel intervention approaches to truly understand efficacy [5–8]. There was great variety in the methods utilized to capture adherence to the intervention across studies limiting comparability as well as ability to assess how the dosage of the intervention received effected the primary outcome of interest. To move the field of IF interventions forward, it is essential to understand how best to implement and disseminate IF interventions in pediatric cohorts. Thus, not only is adherence monitoring required but also the personnel required for adherence monitoring, fidelity training, and prevention of intervention drift to ensure sustained engagement overtime.

Despite the limitations described above, the preliminary efficacy discussed in the reviewed articles exploring the effects of IF on weight loss and cardiometabolic outcomes is consistent with findings reported in adult cohorts [13,14,31]. Despite the diversity in participant demographics and IF strategies—including varying time-restricted eating windows and intermittent energy restriction combined with exercise or supplementation—the research indicates IF, especially TRE, can significantly improve weight loss, body composition, and metabolic health, with potential benefits against metabolic syndrome and type 2 diabetes in adolescent and emerging adult cohorts. However, due to heterogeneity in methodology and quality of the evidence it is challenging to compare the efficacy across studies. Moreover, in adults IF interventions have been shown to have positive effects across other clinical outcomes such as aging, oxidative stress, and inflammation. The current results show the gap in mechanistic data that is available on how IF interventions effect other complex clinical outcomes that may have significant relevance to the long-term benefits of this novel approach [137,138].

Finally, this review draws attention to both the gaps in research regarding the use of IF in adolescents and emerging adults and the opportunities. Expanding diverse nutrition interventions that are developmentally appropriate, practical, and easy to implement across communities and age groups is essential. IF uniquely allows individuals to maintain control over their food choices within a specified eating window. This flexibility in choosing foods, selecting an appropriate eating window, socializing during meals, and dining out without dietary restrictions distinguishes IF as a dietary strategy that fosters sustainable behavioral change for an age group in which autonomy is expanding [18,31]. Given that adolescence is a period of growing independence, reflected in food choices and time management [139], further research is needed to understand adolescent and emerging adult eating patterns and frequencies and how those patterns may affect intervention implementation and dissemination.

The practical implications of the findings from this scoping review on intermittent fasting among youth are significant for parents, educators, and healthcare providers. These stakeholders play a crucial role in shaping the health behaviors of young individuals. By understanding the potential benefits and considerations of IF based on current research, they can better guide and support youth in making informed decisions about their dietary practices. The collective insight from the current review calls for a refined understanding of how IF interventions are designed and implemented in this age group to best accurately capture feasibility and efficacy.

### Strengths and Limitations

To our knowledge, this is the first review of IF evidence among adolescents and young adults. The summary of available studies’ methodology, intervention parameters, outcomes selected, feasibility and efficacy fill an important gap in informing future research priorities. While comprehensive in its scope, the review also has several inherent limitations that could influence the interpretation and applicability of its findings. First, this review's ability to draw generalizable conclusions is challenged by the inherent heterogeneity in design, duration, sample size and characteristics, and methodologies. This variability hinders the broad picture interpretation of IF's efficacy. Particularly concerning is the lack of consistency is capturing intervention adherence. Dosage of the intervention is directly associated with efficacy and thus must be included to ensure efficacy accurately reflects the effect of the intervention. The short duration of many studies on IF involving adolescents and emerging adults, limits the understanding of IF's long-term effects on growth, development, and overall health in this demographic. Additionally, the potential for publication bias, where studies with positive or significant results are more likely to be published than those with negative or inconclusive findings, could inadvertently skew the review's findings in favor of IF. The exclusion of grey literature and non-English texts may further introduce bias, potentially overlooking relevant findings not captured in the mainstream or English-speaking research community. Moreover, the review's approach did not extend to quantifying the quality of reporting or to an in-depth exploration of the methodological quality of the included studies, leaving a gap in our comprehension of the strength and reliability of the evidence base. Together, these limitations highlight critical areas for improvement in future research, underscoring the need for more rigorous, comprehensive, and long-duration studies to fully understand IF's impact on youth.

## Conclusion

In conclusion, our scoping review of 39 studies on intermittent fasting among adolescents and emerging adults highlights significant variability in methodologies, intervention components, feasibility measures, and adherence monitoring, which complicates the assessment of study quality and comparability. This review underscores the need for rigorous studies using standardized theoretical frameworks for acceptability and feasibility to enable comparability across studies and cohorts. This is crucial to determine the practicality and sustainability of IF interventions in this age group. Further research, especially long-term studies, is essential to better understand IF's impact on youth, develop standardized methodologies, and ensure protocols that promote adherence and confirm clinical efficacy.

## Figures and Tables

**Table 1.** Overview of intermittent fasting studies among adolescents and young adults that included efficacy data cataloged by study characteristics, study design, and outcomes.

Reference	Study Design	Participants (sample size; age=mean (SD); % F or M; baseline weight status)	Intervention	Eating window	Multi-component	Duration (days)	Primary Outcome	Secondary Outcome
[31]	RCT	N=45; age=16.4(1.3); 64% females; median weight=101.4 (87.9–123.8) kg; mean BMI z-score= 2.3(0.5)	Arm 1: 8hr TRE + CGM Arm 2: 8hr TRE + blinded CGM Control (12+ hr eating + blinded CGM)	Self-selected eating window	+CGM Low carbohydrate and added sugar	84	Feasibility of TRE	weight loss, dietary intake and quality, physical activity, eating behaviors and practices, and quality of life
[107]	RCT	N=27 with type 2 diabetes; age=16.5(1.7); 63% females; %Body mass index_p95_ = 128.5(28.7)	Arm 1: 8hr late TRE (12:00 – 20:00) Control (12+ hr eating)	Late	+CGM Isocaloric	84	Feasibility	Weight loss, body composition, glycemic control, physical activity, dietary intake, and sleep
[114]	Randomized trial	N=22; age=22(2.5); 100% males; mean weight=90.3(24) kg; mean BMI=28.5(8.3) kg/m2	Arm 1: 8 hr TRE Arm 2: 8hr TRE	Late vs. Self-selected	Isocaloric	28	Cardiometabolic health markers and antioxidant status	Caloric intake
[113]	RCT	N= 60; mean age= 23(0.5); 55% males; mean weight=80(3.5) kg; mean BMI= 27.8(0.8) kg/m^2^	Arm 1: 6hr eTRE (7:00 a.m.–1:00 p.m) Arm 2: 6hr lTRE (12:00 p.m.–6:00 p.m.) Control (ad libitum)	Early vs. Late		56	Body weight and cardiometabolic outcomes	Compliance and feasibility
[112]	Uncontrolled trial	N=33; mean age=22.5 (2.8); 76% females; mean BMI = 22.7(2.7) kg/m2	Arm 1: 8hr TRE	Self-selected		24	Body weight and cardiometabolic outcomes	Meal patterns, sleep, and physiological factors
[115]	RCT	N=16 cyclists; 100% males; mean age= 19.3(0.1); mean weight= 69.66 (6.11); mean BMI=22.16 (1.74)	Arm 1: 8hr TRE (10–11 am to 6–7 pm) Control: Normal Diet (ND) (7 am to 9 pm)	Late		24	Body weight, body composition, and peak power output	Blood biomarkers
[111]	RCT	N=80 active females; 100% males; mean age= 22.1 (2.1); mean weight=76kg; mean BMI=25.14	Arm 1: 8hr TRE from 19:30 to 03:30 Control: Non-TRE	Late		24	Cardiometabolic outcomes and circadian rhythm	Microbial diversity
[110]	RCT	N=40 active females; 100% females; mean age= 22.1(2.6); mean weight=63.9(7.8) kg	Arm 1: 8hr TRE between 12–8 pm + RT Arm 2: 8hr TRE between 12–8 pm and RT Control: diet and RT	Late	β-hydroxy β-methylbutyrate (HMB) measurement Resistance training	56	Body composition	Muscular performance and physiological outcomes
[109]	RCT	N=18 active males; 100% males; mean age=22.45(3.25); mean weight=83.2(16.3) kg	Arm 1: 4hr TRE anytime between 4 pm – 12 am for 4 days/week + RT on the other 3 days of the week Control ND + RT 3 days/week	Late	Resistance Training	56	Body composition	Dietary intake
[108]	Uncontrolled trial	N=22; 100% females; mean age = 21.3(1.2); mean weight= 65.1(12.5) kg	Arm 1: 8hr TRE between 12 – 8 pm	Late	Isocaloric	56	Body weight	Adherence, hunger, satisfaction, and fullness
[32]	Uncontrolled trial	N=30; mean age= 15.1(1.4); 83% females; median BMI= 34.9 (27.7–52.4) kg/m2	IER: VLED 600 – 700 kcal/d for 3 d/wk and Prescribed healthy eating plan 4 d/wk	ADF	Energy Restriction	84	Body weight	Body composition, cardiometabolic outcomes, vascular structure, eating behaviors, and quality of life
[116]	RCT	N=34; healthy participants; mean age= 23.9; 50% females, mean BMI= 27.0 kg/m2	Arm 1: continuous energy restriction (CERT group) Arm 2: 5:2 intermittent fasting (IFT group) diet	ADF	Energy Restriction (All participants underwent resistance training (3 sessions per week) and aimed to have 20% energy restriction and ≥1.4 g/kg/day Resistance training	84	Cardiometabolic outcomes	Hunger, craving, and mood
[117]	RCT (Crossover study)	N=17; healthy participants; mean age= 24; mean BMI=~ 24 kg/m2	Arm 1: IF only (IF) Arm 2: IF with antioxidant supplementation (IFAO)		Isocaloric	70	Gene expression	Oxidative stress
[118]	RCT (Crossover study)	N=12; healthy volunteers; 58% females; mean age=24 (2.3); mean BMI= 21.9 (1.71) kg/m^2^	Arm 1: 5.5 TRE Control: 11 hr eating control	Late	Isocaloric	14	Energy balance	Blood glucose and physiological markers
[119]	RCT (Crossover study)	N=12; healthy students; 100% males; mean age= 22.4 (2.8); mean BMI= 24.2 (2) kg/m^2^	Arm 1: 8hr TRE between 1–9 pm Control: Non-TRE control (usual diet w/o time restriction)	Late	Isocaloric	56	Wingate anaerobic test performance	Body composition
[120]	RCT	N=14; lean men; 100% males; mean age= 25 (4); mean BMI= 24 (2) kg/m^2^	Arm 1: Energy Restriction (ER): 24-hr 25% calories based on estimated needs Control: Energy Balanced (EB): 24-hr 100% % calories based on estimated needs	ADF	Energy Restriction	2	Glycemic control	Body mass
[121]	RCT	N=77; college students; 100% females; mean age= 25 (4); mean BMI= 24 (2) kg/m^2^	Arm 1: 8hr TRE Arm 2: Exercise Arm 3: 8hr TRE + Exercise Control	Late	Resistance training	56	Body weight	Body composition and lipid levels
[122]	Uncontrolled trial	N=34; healthy young adults; 64.7% females; mean age= 23.4 (2.9); mean BMI = 24 (2) kg/m^2^	Arm 1: 8hr TRE + snack packages with 20 g protein / day	Self-selected	Isocaloric	24	Body composition	Cardiometabolic outcomes
[123]	RCT	N=26; recreationally active males; 100% males; mean age=~22.7; mean weight=~82.65 kg	Arm 1: 8hr TRE with 25% calorie deficit Control: normal diet with 25% calorie deficit		Energy restriction Resistance training	24	Body composition	Muscle performance, resting energy expenditure, and blood biomarkers
[124]	RCT	N=20; healthy lean subjects; 100% males; mean age=23.5 (2.8); mean weight= 78.7 (8.5) kg	Arm 1: Alternate Day Calorie Arm 2: Restriction (ADCR): bed rest with 25% of total energy requirements every other day and 175% of total energy requirements every other day Control: bed rest with three daily isoenergetic meals	ADF		8	Insulin resistance	Cognitive function, body composition, and physical capacity
[125]	Uncontrolled trial	N=21; 76.2% females; mean age=16.2 (1.4); mean BMI= 41.9 (6.2) kg/m^2^	Arm 1: Protein-sparing modified fast (PSMF): 1200–1800 calories, 40–60 g of carbohydrate/day and 1.2–1.5 g protein/kg of ideal body weight	PSMF	Energy restriction	365	Body weight	BMI and psychosocial HRQOL
[126]	RCT	N=29; 41.4% females; mean age=22 (3.34); mean BMI= 419 (6.2) kg/m^2^	Arm 1: 8hr TRE Control: eating an 810–860 kcal meal 4–5 hrs before each study visit	Late		5	Muscle damage indicators	Inflammation and oxidative stress markers
[127]	RCT (Crossover study)	N=24; healthy lean; 50% females; mean age= 23(2.6); mean BMI= 22.1 (2.5) kg/m^2^	Arm 1: 12hr overnight fast Arm 2: 14hr overnight fast Arm 3: 16hr overnight fast			9	Postprandial glycemia and insulinemia	Energy intake at subsequent meal
[131]	RCT (Crossover study)	N=12; healthy males; 100% males; mean age= 25(3); mean BMI= 26 (4) kg/m2	Arm 1: FASTED AM-brisk walking Arm 2: FED AM-brisk walking Arm 3: FASTED PM-brisk walking Arm 4: FED PM-brisk walking	Early vs. Late	brisk walking	4	Gastric emptying	Metabolic responses and appetite
[130]	RCT	N=20; healthy college students; 100% males; mean age= 20.5(1); mean Weignt= 67 (4.5) kg	Arm 1: intermittent fasting (four meals) Control: five meals		Calorie restriction and high-intensity exercise	10	Wingate Anaerobic Power and High-Intensity Time-to-Exhaustion (HIT) Cycling Performance	Physiological Measures
[129]	RCT (Crossover study)	N=8; active males; 100% males; mean age= 25(2); mean Weight= 74.6 (5.2) kg	Arm 1: fasted state cycling Control: fed state cycling (consumed a carbohydrate-rich mixed- macronutrient breakfast 2 hours before exercise)		cycling	2	Skeletal muscle signaling responses	Substrate Availability and Utilization
[128]	RCT (Crossover study)	N=12; young males; 100% males; mean age= 21(0.5); mean BMI= 22.5(1.7) kg/m2	Arm 1: Energy restriction condition (Def-EI) involving a 24-hour fast. Arm 2: Exercise condition (Def-EX) with energy depletion matched to the energy restriction condition through exercise. Control: no energy depletion		Energy restriction and exercise	6	Appetite and energy intake responses	Macronutrient intake and food reward
[105]	RCT (Crossover study)	N=21; healthy young adults; 71% males; mean age= 21(3); mean BMI= 25(3) kg/m2	Arm 1: short-term energy deprivation (ED) Control: energy balance (EB)		Energy restriction	4	Insulin, Acyl Ghrelin, and Leptin	Appetite and energy intake
[95]	RCT	N=50; adolescents with obesity; 72% females; mean age= 16.4(1.3); mean BMI z-score= 2.3(0.5)	Arm 1: 8hr TRE + CGM Arm 2: 8hr TRE + blinded CGM Control: (12+ hr eating + blinded CGM)	Self-selected eating window	+CGM Low carbohydrate and added sugar	84	Feasibility of CGM wear, glycemic profiles, and glycemic excursions	Weight change

**Table 2.** Overview of intermittent fasting studies among adolescents and young adults that included efficacy data cataloged by results reported.

Reference	Feasibility/Acceptability	Adherence	Weight outcome	Metabolic markers	Biological markers
[31]	Satisfaction Questionnaire, and exit Interview: 90% reported that the study was worthwhile, 95% reported that they would recommend it to others, 15% reported barriers to implementing their assigned eating window, mean score of how helpful TRE was = 4/5, mean score of how enjoyable TRE was = 4/5	HOW: Adolescents were asked to record the time they started and finished eating daily, the number of days they adhered to their prescribed eating schedule, and barriers to adherence. RESULTS: Mean number of TRE compliant days = 5.2 d/week, 15% reported barriers to implementing their assigned eating window including conflict with work or sleep schedule, social commitments, and explaining eating patterns to family.	^-^^3^5% weight loss: -in 31% of TRE+CGM -in 26% of TRE+blinded CGM -in 13% of the control group	N/A	N/A
[107]	HOW: participant satisfaction Satisfaction surveys showed that lTRE was viewed favorably by most participants.	HOW: recording the time, they started and finished eating daily, the number of days they adhered to their prescribed eating schedule, and barriers to adherence. RESULTS: Adherence to the lTRE protocol was high, with mean compliant days = 6.2 d/week, 3/27 adolescents reported barriers to implementing their assigned eating window into their daily schedule, including conflict with work or sleep schedule, social commitments, and explaining eating patterns to family	- %BMI_p95_ at week 12 by: –3.4% in the lTRE group and –2.8% in the control ^-^ %BMIp95 at week 12 by 5% in: 46% of the lTRE and 21% in control	^-^ HbA1C in both groups C-peptide in lTRE ^-^ALT in lTRE	N/A
[114]	Not specifically measured in this study.	Participants adhered to the 16:8 time-restricted feeding protocol consistently, with an average eating window of 7.2 ± 0.7 hours and an average fasting time of 16.7 ± 0.8 hours	^-^ body mass (kg) in both groups	HDL in both LDL in ad libitum > isocaloric TC in ad libitum > isocaloric cortisol in ad libitum > isocaloric insulin in ad libitum > isocaloric RMR in ad libitum > isocaloric FM in ad libitum > isocaloric ^-^ FM in both groups ^-^ SBP in both groups ^-^ DBP in both groups ^-^ HR in both groups	adiponectin in both CRP in ad libitum > isocaloric glutathione in ad libitum < isocaloric NOx in ad libitum > isocaloric
[113]	Not specifically measured in this study.	Both eTRE and lTRE groups were able to adhere to their respective eating windows throughout the 8-week intervention. However, adherence gradually decreased over time.	^-^ weight and BMI in eTRE and lTRE compared to control ^-^ waist circumfrance in eTRE compared to control	^-^ FM in eTRE and lTRE compared to control ^-^ FFM in eTRE compared to control, and in lTRE compared to eTRE ^-^ FM% in lTRE compared to control ^-^ visceral fat in eTRE and lTRE compared to control ^-^ mean BG in eTRE compared to control mean BG in lTRE compared to eTRE ^-^ C-peptide, insulin resistance(HOMA-IR & fasting insulin), and SBP in eTRE compared to control ^-^ leptin in eTRE and lTRE compared to control LDL in eTRE and lTRE compared to control	superoxide dismutase (SOD) in eTRE compared to lTRE and control
[112]	Not specifically measured in this study.	Participants maintained an average of 2.8 eating occasions per day. Meal patterns showed consistent adherence, with participants consuming 39.2% of energy at lunch, 37.6% at dinner, and 18.5% via snacks. Despite high adherence, the weight gain group consumed significantly more saturated fat at dinner (6.0% of total energy, p = 0.0241) compared to the weight loss group (3.1% of total energy).	in total group and female subgroup but not male subgroup: ^-^ mean weight ^-^ mean BMI	^-^ mean FM% in total group ^-^ fasting insulin in the subgroup who lost weight ^-^ HOMA-IR in the subgroup who lost weight LDL in total group and in the subgroup who lost weight ^-^ HDL in the subgroup who lost weight	N/A
[115]	Not specifically measured in this study.	All participants followed their prescribed diet plans, which included regular meal timing, caloric intake, and macronutrient distribution.	^-^ body weight of 2% in TRE compared to baseline	FM% of ND at week 4 > FM% of TRE at week 4 ^-^ IGF-1 and testosterone free in TRE (but FFM was maintained) ^-^ cortisol in both groups	adiponectin/FM in TRE compared to baseline ^-^ leucocytes in ND ^-^ neutrophils% in both groups lymphocytes in both groups ^-^ neutrophil-to-lymphocytes ratio in TRE (infiammatory marker)
[111]	Not specifically measured in this study.	Not specifically measured in this study.	N/A	^-^ TC and TAG in post TRE compared to pre-TRE and to non-TRE level. LDL remained the same HDL in post-TRE compared to non-TRE ^-^ AST and ALT in post TRE compared to pre-TRE and non-TRE	serum IL-1B and TNF-a reduced in post-TRE but not statistically significant
[110]	HOW: questionnaires RESULTS: At 4 weeks, 84% of participants reported no side effects. At 8 weeks, 90% of participants reported no side effects. REPORTED SIDE EFFECTS included: •Suppressed appetite (TRF group: 1 participant) •Increased appetite with irritability (TRF group: 1 participant) •Morning fatigue (TRFHMB group: 1 participant) •Nausea (CD group: 1 participant) •Bloated stomach (CD and TRFHMB groups: 1 participant each) QUESTIONNAIRE RESPONSE: •Improvements in scores for the Mood and Feelings Questionnaire at W4 and W8 compared to baseline (W0) in all groups. •Reduction in the uncontrolled eating score of the Three Factor Eating Questionnaire across time in all groups. •Proportion of participants with regularly occurring menstrual cycles ranged from 69% to 79% across groups.	Compliance with the assigned eating schedule was ≥89% on average.	weight in all groups	FFM and muscle thickness in all groups ^-^ FM and FM% in TRE and TRE+ HMB groups but in control	N/A
[109]	reported Difficulty of TRF (VAS) 4 weeks: 3.6 ± 1.4 out of 10 8 weeks: 3.8 ± 2.2 out of 10 (p = 0.86)	Compliance Rates TRF Programme: 95.9 ± 4.1% RT Programme (RT-ND): 92 ± 10% RT Programme (RT-TRF): 91 ± 8%	no significant change	no significant change in FFM, FM, or FM%	N/A
[108]	Self-reported measures of hunger, satisfaction, and fullness remained stable over the 4-week period (0: “Not hungry at all, completely empty, or not full at all” and 100: “Never been more hungry, cannot eat another bite, or totally full”: Hunger: average 45 mm (p = 0.877) Satisfaction: average 51 mm (p = 0.589) Fullness: average 51 mm (p = 0.812)	HOW: Weekly emails were sent to participants reminding them to record their adherence, hunger, satisfaction, and fullness. Participants reported high adherence, maintaining the eating regimen for an average of more than 5.5 days per week with no significant variations across the weeks (p = 0.902)	^-^ body weight with mean change of −0.6 kg	^-^ FM in the subgroup performing resistance exercise, but this wasn’t reported in the other subgroups (nonexercised, endurance-exercise, or endurance-resistance-exercise)	N/A
[32]	Participants rated the IER diet as easy and pleasant to follow (mean ± SD on a Likert scale from −4 to +4: +2.1 ± 1.2 for ease, +1.9 ± 1.2 for pleasantness). At 12 weeks, 23 participants chose to continue with IER, and at 26 weeks, 19 participants remained on the VLED.	Participants maintained adherence to the VLED on prescribed days, with reported energy intake closely aligning with the prescribed 2500–2900 kJ/day	^-^ BMI %95th ^-^ BMI z-score ^-^ waist-to-height ratio	No changes	N/A
[116]	EASE OF COMPLIANCE: IFT Group: Mean score 6.6 ± 1.8 out of 10 CERT Group: Mean score 7.1 ± 1.3 out of 10 Between Group: Not significant (p = 0.36) PREFERENCE FOR DIET GROUP: IFT Group: 4 participants would have preferred to be in the CERT group CERT Group: 3 participants would have preferred to be in the IFT group CONTINUATION OF DIET POST-INTERVENTION: IFT Group: 14 participants indicated they would continue with the diet, but only 1 would continue as prescribed. CERT Group: 15 participants indicated they would continue with the diet, but only 2 would continue as prescribed	DIETARY COMPLIANCE: The study reported high rates of dietary compliance (80%) in both the IFT and CERT groups.	N/A	^-^ TC in the IFT group more than the CERT group ^-^ LDL in the IFT group more than the CERT group	no significant difference in high-sensitivity CRP
[117]	HOW: Dietary satisfaction survey RESULTS: A majority of participants expressed difficulty with finishing food on feasting days (76% agreed) and with eating so little on fasting days (65% agreed). Comparing fasting and feasting days, the majority of participants disagreed that feasting was more difficult than fasting (55% disagreed). Few participants rated the diet as improving quality of life overall (35% agreed). Importantly, a majority of participants rated the fasting days as making daily activities more difficult, but less than half rated the feasting days as making daily activities more difficult. Although participants would recommend the diet to a friend (71% agreed), most described the diet as more difficult than previously attempted diets (71% agreed), and only 18% would follow the diet if prescribed by a physician.	High compliance was noted, with participants consuming within 5% of the prescribed caloric intake on most pre-conditioning and fasting days. However, there was lower adherence on feasting days, with significant difficulty in consuming the full 175% of caloric intake.	The study aimed to maintain weight.	^-^ plasma insulin in IF group (0.0023) but not the IFAO group (0.33)	(not significant but a trend) expression of *SIRT3* in the IF (p=0.0772)
[118]	The study did not explicitly measure feasibility.	The participants adhered strictly to the dietary intervention protocol, which involved consuming an isocaloric diet under rigorous control conditions. All food intake, physical activity, and other variables were meticulously monitored to ensure compliance with the intervention protocol.	N/A	fecal energy loss and a (non-significant) trend in urine energy loss without energy expenditure alteration in TRE compared to control ^-^ energy balance in TRE compared to control ^-^ mean 24-hour BG and heart rate in TRE compared to control	N/A
[119]	The study did not specifically measure feasibility.	Adherence to the dietary interventions was assessed through self-reported dietary records. Despite these measures, six participants were excluded for non-adherence.	No significant change	No significant change in body composition	N/A
[120]	The study did not specifically measure feasibility.	Adherence was assessed indirectly through controlled provision of meals and instructions for consumption. Compliance was ensured by providing all food and drink during the study period and instructing subjects to perform minimal activity.	^-^ body weight in both groups but significantly to a greater extent after ER	^-^ fasting BG after ER compared to EB ^-^ fasting serum insulin and HOMA2-IR after ER compared to EB fasting NEFA after ER compared to compared to EB postprandial BG after ER compared to EB postprandial serum insulin after ER compared to EB postprandial NEFA after ER compared to EB	N/A
[121]	The study did not specifically measure feasibility.	Dietary adherence was not evaluated. Minor adverse reactions were reported, including lack of concentration, dizziness, tiredness, thirst, irritability, leg pain, and back pain.	^-^ body weight and lean body mass in TRE, TRE+Exercise, and Exercise groups compared to baseline ^-^ body weight in TRE and TRE+Exercise groups compared to control ^-^ BMI in TRE, TRE+Exercise, and Exercise groups compared to control	fat% in Exercise and TRE+Exercise groups compared to baseline ^-^ lean body mass in TRE, TRE+Exercise, and Exercise groups compared to control fat% in Exercise and TRE+Exercise groups compared to TRE group TC in TRE, TRE+Exercise, and Exercise groups after study period	N/A
[122]	The study did not specifically measure feasibility.	only one participant lost to follow-up Participants maintained their self-selected eating windows, and adherence was monitored through daily dietary records and sleep logs	^-^ body weight and BMI only in the early TRE group compared to baseline ^-^ waist circumference in all participants	^-^ FM and FM% only in the early TRE group compared to baseline ^-^ fasting BG, HOMA-IR, insulin, TG, and HDL in the early TRE group compared to baseline LDL HDL in the early TRE group compared to baseline ^-^ TG in the late TRE group compared to baseline	N/A
[123]	HOW: The perceived recovery scale (PRS), daily analyses of life demand for athletes (DALDA), and visual analogue scales (VAS) RESULTS: No significant differences between the groups were noted for emotional eating, uncontrolled eating, perceived recovery between training sessions, VAS (energy, desire to eat, fullness, hunger, and motivation to do physical tasks), or perceptions of daily life stressors	Participants reported dietary intake for three days each week using the MyFitnessPal application. A member of the research team checked food log compliance each week during resistance training sessions. Total calories, relative calories, carbohydrate (grams), fat (grams), and protein (grams) were collected from food logs. Average macronutrient and calorie intakes were compared between groups.	^-^ body weight, FM, and FM% in both groups	^-^ testosterone and REE in both groups plasma cortisol levels only in control	^-^ adiponectin in both groups
[124]	Not specifically measured or reported.	The mean recorded daily energy intake was slightly lower than the estimated daily energy expenditure (7.4 kcal/day deficit, 95% CI: 2.1; 12.6, P = 0.009). The ADCR group managed to overeat every other day to compensate for the days of caloric restriction, ensuring overall energy balance.	^-^ body weight among all participants compared to baseline	No positive effects were associated with ADCR on the negative health outcomes of bed rest. cortisol among all participants compared to baseline total cholesterol and LDL among all participants compared to baseline ^-^ HDL among all participants compared to baseline	TNF-a among all participants compared to baseline
[125]	Not specifically measured or reported.	Adherence was measured based on participants’ dietary recalls and categorized as “adherent” or “non-adherent.” At 3 months, adherence was higher among participants who attended clinic visits (58.3%). Adherence dropped significantly by 6 months (37.5%).	^-^ weight after 6 months (−4.7(6.6) kg; p=0.04) but nonsignificant after 12 months (−1.3(10.6) kg; p=0.35)	N/A	N/A
[126]	Not specifically measured or reported.	Participants adhered to the fasting and dietary restrictions, as verified by questionnaires.	N/A	N/A	NO in fasting compared to control group ^-^ TNF-a in fasting compared to control group
[127]	Not specifically measured or reported.	Not specifically measured or reported.	N/A	^-^ fasting BG and fasting insulin in all participants postprandial glycemic response in females with longer fasting periods ^-^ HOMA-IR in females as fasting increased from 12 to 16 hours	N/A
[131]	Not specifically measured or reported.	Not specifically measured or reported.	No significant differences in weight were reported.	No significant differences were reported for glucose concentrations. Significant trial effect for CHO oxidation, with higher rates in FED trials (P = 0.001).	N/A
[130]	Not specifically measured or reported.	Not specifically measured or reported.	No significant differences in weight were reported.	Significant effect of the fasting group on reducing triglycerides was reported (p<0.0001).	N/A
[129]	Not specifically measured or reported.	Not specifically measured or reported.	N/A	No significant differences in weight were reported.	N/A
[128]	Not specifically measured or reported.	The study design involved healthy young males and the adherence to the protocol was ensured by the controlled laboratory settings and the randomization process. Participants adhered to the dietary restrictions or usual dietary habits as per the protocol.	N/A	N/A	N/A
[105]	Not specifically measured or reported.	21 out of 31 enrolled participants completed both ad libitum meal tests. Reasons for attrition included relocation, unrelated illness, esophageal reflux, nausea/vomiting, weakness and hunger, and inability to provide blood samples. Non-completers did not significantly differ from completers in age, sex, or BMI.	Participants experienced a significant reduction in body weight during energy deprivation (p<0.001).	Fasting Glucose decreased significantly during ED but remained unchanged during EB (p<0.001). Fasting Insulin decreased significantly during ED but remained unchanged during EB (0.01).	N/A
[95]	Not specifically measured or reported.	The study did not report adherence to TRE, but the results reported high adherence to daily CGM wear (96.4%) without negative impacts on daily functioning.	No significant association between change in weight and fasting glycemic excursions (correlation coefficient = 0.19, p = 0.3).	All data related to glycemic profiles and excursions were obtained from continuous glucose monitoring devices. No significant change in glycemic variability, as measured by standard deviation, mean amplitude glycemic excursion (MAGE), and glucose area under the curve (AUC), was observed over the study period between the intervention and control groups.	N/A

## Abbreviations

BMI: Body mass index
zBMI: Body mass index Z-score
%BMI_p95_: Percent over the 95th percentile
IF: Intermittent Fasting
TRE: Time Restricted Eating
ADF: Alternate Day Fasting
